# Cerebral metastasis from anal squamous cell carcinoma: A case report and literature review

**DOI:** 10.3892/ol.2025.15086

**Published:** 2025-05-13

**Authors:** Elena Andreea Popa, Vanesa Marisa Tomatis, Esther Quick, Paul Stephen Mitchell, Chrisovalantis Tsimiklis, Annika Reann Mascarenhas

**Affiliations:** 1 Department of Neurosurgery, Flinders Medical Centre, Adelaide, South Australia 5042, Australia; 2 Department of Molecular Pathology, SA Pathology, Adelaide, South Australia 5042, Australia

**Keywords:** brain metastasis, anal cancer, squamous cell carcinoma

## Abstract

Anal cancer comprises only 3% of all gastrointestinal malignancies, of which only a small proportion of patients will experience distant metastasis; very few of these cases will metastasise to the brain. The present case report details the clinical course of a 56-year-old female patient who was previously diagnosed with anal squamous cell carcinoma (SCC) and subsequently developed an isolated cerebral metastasis ~5 years later. The patient was initially diagnosed with anal SCC after presenting with a non-tender lump in the groin and had no other signs or symptoms. After undergoing a lumpectomy in 2017, the patient was lost to follow-up. In 2022, the patient presented with a perforated rectal tumour and consequently underwent a loop colostomy followed by chemoradiotherapy. The patient was considered to be in remission at the end of 2023 until subsequent presentation months later with vague neurological symptoms, which led to the diagnosis of a large metastatic lesion in the right temporal lobe. Following resection via craniotomy and additional radiation therapy, there was no evidence to suggest recurrence of either the anal primary tumour or cerebral metastasis from follow-up imaging 5 months post-operatively. The present report highlights the potentially aggressive nature of anal SCC, late-onset cerebral metastasis and the need for further investigation into standardising treatment protocols and surveillance strategies for gastrointestinal metastatic disease.

## Introduction

Anal cancer is a rare but serious malignancy accounting for ~3% of all gastrointestinal types of cancer (1). Squamous cell carcinoma (SCC) is the most common type of anal cancer and is suspected to be linked to inflammatory processes secondary to human papilloma virus (HPV), particularly subtypes 16 and 18 (2). A third of anal cancers are asymptomatic or present with non-specific symptoms, while the remaining cases will present with anal bleeding or pain (3). Anal cancer treatment is multi-disciplinary and stage dependent, consisting of combined chemoradiotherapy (CRT) and/or surgical resection (2). The 5-year overall survival of localized disease is ~78%, and 18% in metastatic disease, with the median survival of metastatic disease being 12 months (4). In anal SCC, distant metastasis is relatively uncommon, with the liver being the most common area for metastatic spread (4). Distant metastasis of anal SCC has been reported in 5–8% of cases at diagnosis, with 10–20% after curative local treatment (5). An even more uncommon occurrence of anal SCC is metastatic spread to the brain; to the best of our knowledge, only 9 previous cases have reported cerebral metastatic disease after an initial anal SCC diagnosis (6–13). The current study presents the case of a 56-year-old female with anal canal SCC who later developed cerebral metastasis, adding to the limited literature of this rare phenomenon.

## Case report

The current study presents the case of a now 56-year-old right-handed Caucasian female who initially presented with anal SCC (T3 N1c Mx) and later went on to develop an isolated cerebral metastasis. In September 2017, the patient presented to a rural hospital in South Australia with a non-tender lump in the right pubic area with no reported systemic symptoms. A fine-needle aspiration under ultrasound guidance demonstrated malignant cells, in keeping with poorly differentiated SCC that was reactive for p16, a tumour marker highly associated with HPV (14). A computed tomography (CT) scan of the abdomen and pelvis demonstrated no primary tumour. The patient underwent pubic lumpectomy in February of 2017 and was subsequently offered combination CRT but was lost to follow-up due to scheduling difficulties.

In September 2022, the patient presented to the Emergency Department of a local rural hospital with sharp anal pain and obstipation, in which a CT scan demonstrated a perforated rectal tumour above the anal verge. The perforation was repaired, and the tumour was removed laparoscopically via loop colostomy in November 2022 following a multi-disciplinary team meeting. The subsequent pathology report confirmed the presence of invasive basaloid SCC with focal necrosis, in keeping with the initial primary diagnosis in 2017. The tumour expressed p40 positivity, which indicated SCC origin (15). Concurrent CRT with mitomycin-C with oral capecitabine was commenced, and the patient also underwent 54 Gy of targeted radiation therapy in 27 fractions. The patient was declared in remission in December of 2023 and shortly thereafter underwent a reversal of the colostomy the following month.

**Figure 1.**
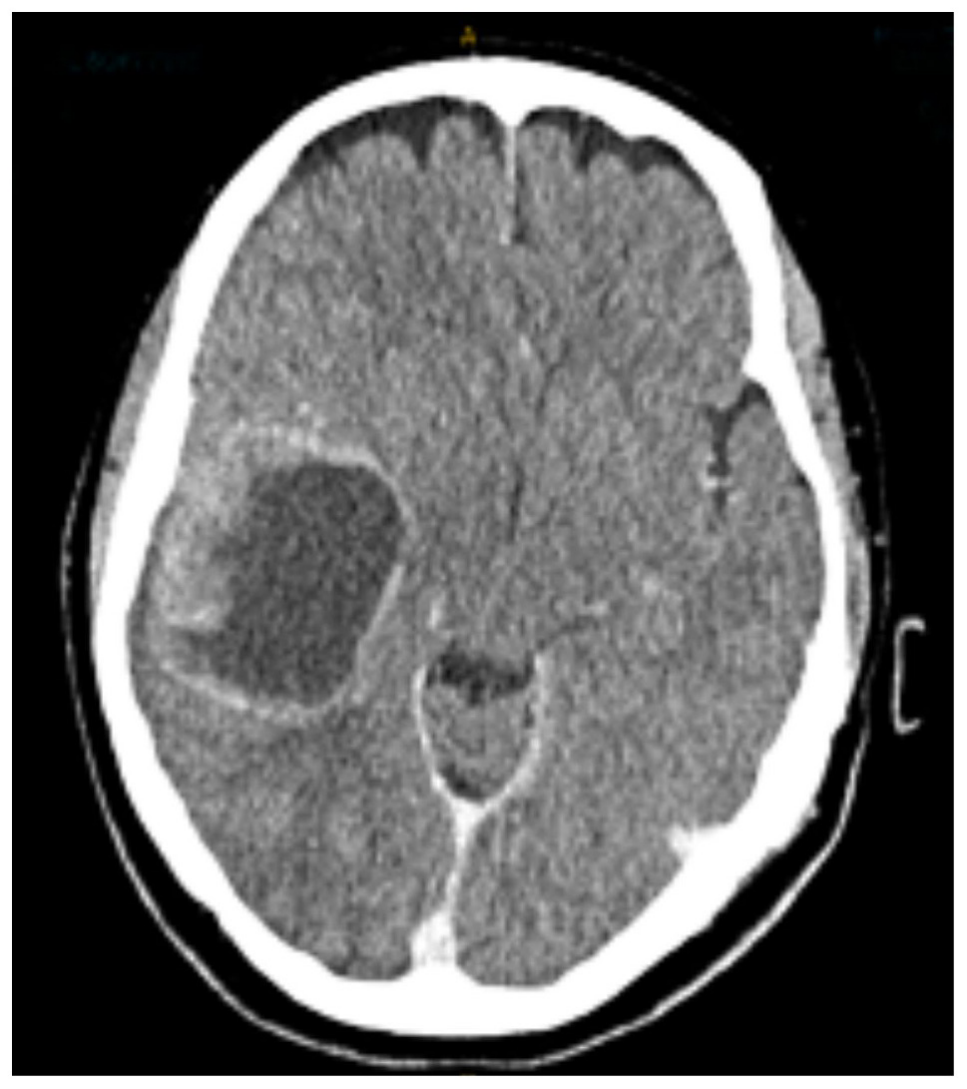
Post-contrast axial CT scan demonstrating large peripherally enhancing heterogenous solid-cystic tumour with localised mass effect. CT, computed tomography.

The patient presented to the Emergency Department in March 2024 with several weeks of lethargy, disorientation, labile mood, personality changes and headaches. Physical examination was unremarkable on admission. A CT scan demonstrated a 5.2×4.2×4.9 cm rounded solid and cystic, irregular enhancing mass in the right frontotemporal region of the brain, in keeping with metastasis (Fig. 1). There was midline shift to the left, effacement of the right lateral ventricle and mild perilesional oedema. The patient was subsequently transferred to Flinders Medical Centre (Adelaide, Australia), and admitted for review and treatment under the Neurosurgical Unit.

**Figure 2.**
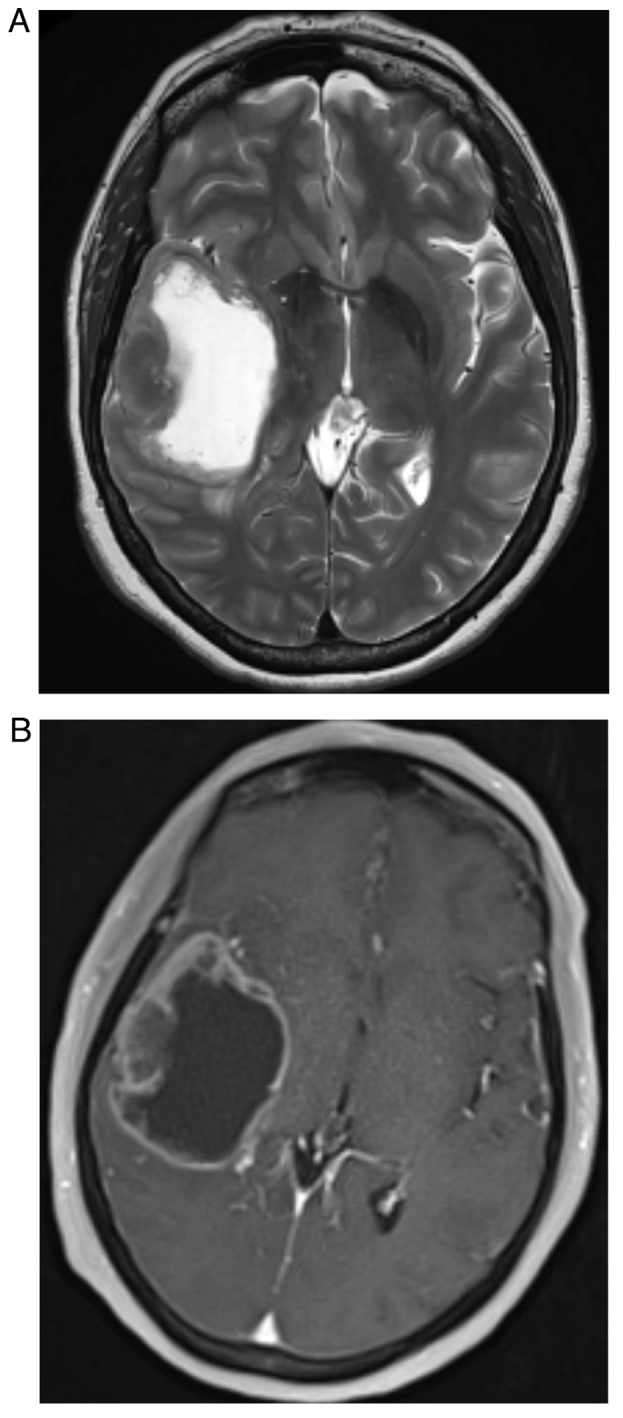
T1 and T2 MR images demonstrating the metastatic tumour. (A) T2 MRI sequence demonstrating high signal intensity within a cystic centre surrounded by thickened heterogenous rim of tumour and mild perilesional change. (B) Post-contrast T1 MRI sequence demonstrating heterogeneously peripheral rim enhancing tumour. MRI, magnetic resonance imaging.

CT scans of the chest, abdomen and pelvis did not demonstrate recurrent colorectal malignancy or new nodal or metastatic disease. Magnetic resonance imaging (MRI) of the brain with gadolinium confirmed a large cystic and necrotic solitary metastatic lesion within the temporal lobe (Fig. 2).

**Figure 3.**
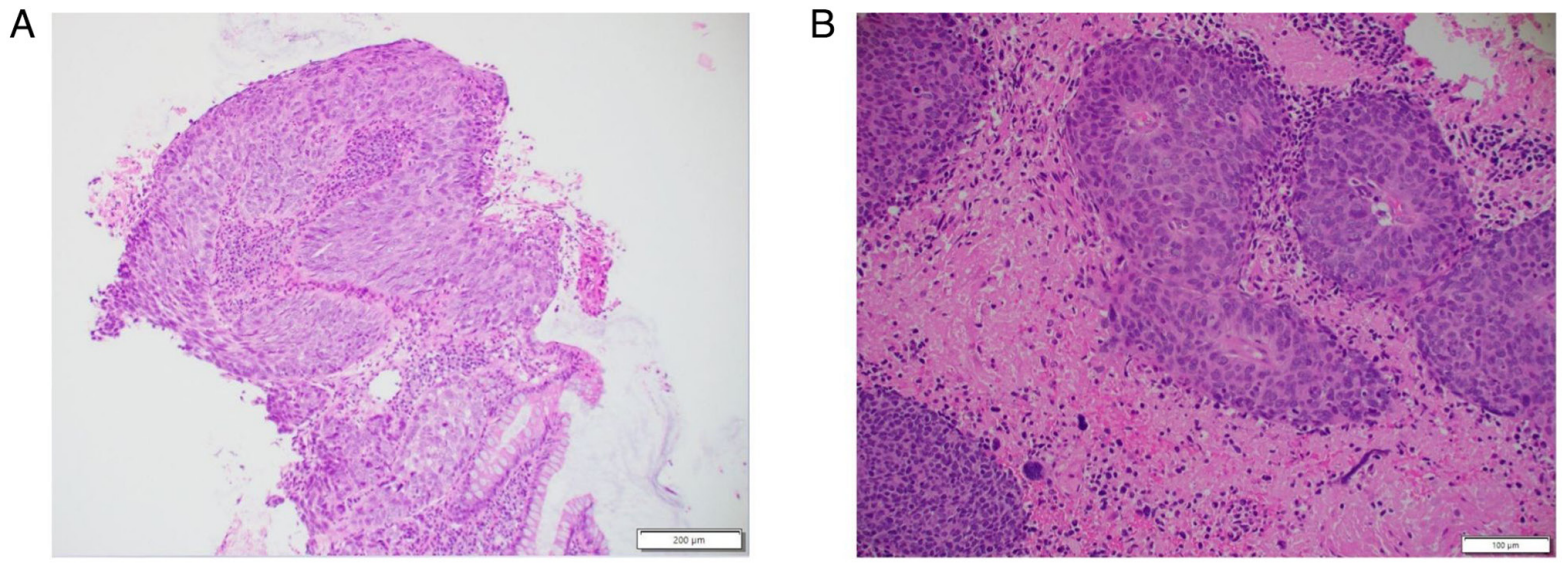
Representative haematoxylin and eosin-stained sections. Stained sections demonstrating squamous cell carcinoma in the (A) primary anal tumour (scale bar, 200 µm) and (B) metastatic brain lesion (scale bar, 100 µm). The sections show malignant epithelial cells with nuclear enlargement, hyperchromasia, pleomorphism, mitotic activity and focal keratinisation.

**Figure 4.**
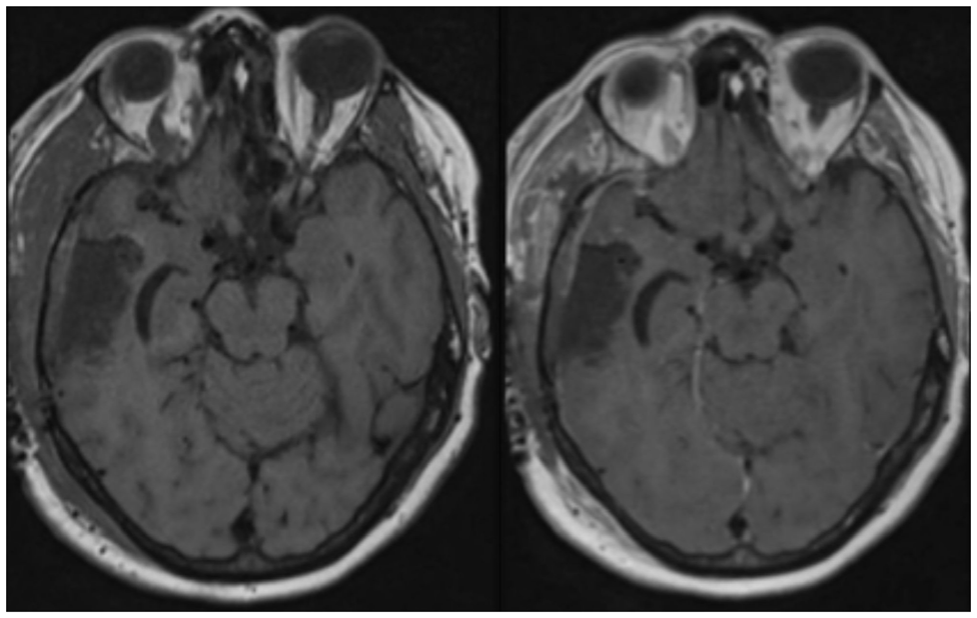
Day 1 post-operative axial pre- and post-contrast T1-weighted MRI showing no significant residual tumour post-craniotomy and resection.

Following counselling and consent, the patient underwent neuronavigation-guided craniotomy and resection of the tumour in March of 2024. Histopathological analysis of haematoxylin and eosin staining confirmed the presence of cells consistent with sheets and nests of malignant basaloid epithelial cells demonstrating nuclear enlargement, hyperchromasia and marked pleomorphism, as well as brisk mitotic activity in addition to focal keratinisation (Fig. 3). A post-operative MRI did not demonstrate any residual tumour (Fig. 4). The patient had recovered well post-operatively without any complications and was discharged home on day 9 post-operatively, with referrals to the original oncologic team for consideration of further CRT.

The patient attended a follow-up appointment with a medical oncologist 1 month after discharge, who discussed radiation therapy. It was considered that systemic chemotherapy would not have provided any benefit at the time due to the lack of progression of systemic disease. The patient underwent 30 Gy over 10 fractions of hypofractionated radiotherapy to the cerebral resection cavity. The patient had repeat abdomen CT and brain MRI scans (Fig. 4) 4 months post-operatively, which showed no recurrence of either anal primary or cerebral metastasis. At a 2- and 5-month neurosurgical review, no ongoing clinical issues were reported except mild fatigue, and the patient remained neurologically intact. The patient was subsequently discharged from the neurosurgical clinic with no further follow ups.

## Discussion

Anal cancer, a rare but deadly malignancy, is increasingly prevalent in Western countries. SCC is the most common form of anal cancer, accounting for 80% of cases (1). Primary anal adenocarcinoma comprises 10–20% of anal cancer cases, while anal melanoma and basal cell carcinoma are exceedingly rare, seen at 1–4 and 0.2%, respectively (1). Certain risk factors increase the likelihood of developing anal cancer, such as smoking, HIV and sexually transmitted diseases such as HPV subtypes 16 and 18. Once anal cancer has metastasized, it carries a poor prognosis and is often fatal (4). Other risk factors include males who engage in sexual activity with males, receptive anal intercourse, history of other sexually transmitted diseases, use of immunosuppressive medication and women with a history of cervical cancer (16). The incidence of anal cancer has increased in Western countries over the last few decades at ~2.2% per year, likely due to changes in sexual behaviour leading to sexually transmitted infections. Individuals who have undergone organ transplantation are also at an increased risk for anal cancer due to the necessary immunosuppressive medication regimen, allowing cancer cells to proliferate without mounting a significant immune response (16). Combined CRT, commonly utilising 5-fluorouracil (5-FU) in addition to cisplatin or mitomycin-C, is the standard treatment for anal SCC, but there is no current consensus for treating metastatic disease (4).

To date, 9 previous cases of cerebral metastasis from anal SCC have been documented in the literature, with ages ranging from 44–69 years (see Table I) (6–13). Among these cases, 5 patients were female, 3 were male and the sex of 1 patient was not disclosed. Treatment of metastatic brain lesions varied based on the location and extent of the metastasis, the patient's preferences, and their functional status at diagnosis. None of the reported cases initially presented with known metastatic cerebral disease and 3 cases presented with no evidence of metastasis at all (6,11,12). The most common locations for distant metastasis were the local lymph nodes, the liver or both, as observed in 6 patients (7–10,13). Only 2 cases initially presented with lung metastasis at diagnosis (10,13). There was no discernible pattern when comparing the natural history of these cases. The first documented case of cerebral metastatic spread was secondary to anal cloacogenic carcinoma in 1967. No further information was reported regarding patient demographics, staging, treatment or outcome for this patient (12).

In the present study, the patient was diagnosed with anal malignancy at the end of 2017. After being lost to follow-up following their initial presentation, the patient underwent appropriate treatment with CRT in 2022. However, despite undergoing appropriate treatment for anal SCC, the patient was diagnosed with a cerebral metastatic lesion ~6.5 years following initial diagnosis and 6 months after achieving remission. The shortest reported interval between primary and metastatic diagnosis was 3 months (6) and the longest was 8 years (11). Malla *et al* (6) reported a patient to have metastatic spread to the frontal lobe who underwent surgical resection of the brain tumour plus γ knife radiosurgery before experiencing a recurrence of the brain lesion 8 months later and was subsequently treated with whole-brain radiation therapy (WBRT). Despite receiving WBRT, another cerebral recurrence was discovered 7 months later. The patient then opted for hospice care instead of further treatment.

The longest period between primary diagnosis and metastatic discovery was reported by Davidson and Yong (11) in 1991. A 61-year-old female patient with anal SCC presented with neurological symptoms 8 years after undergoing surgical resection of the primary malignancy. The patient subsequently underwent surgical resection and radiotherapy for the brain lesion. This was the only reported case with surgical resection of the primary malignancy, similar to the present case.

Of the 9 reported cases, 2 patients had succumbed to their disease before commencing treatment (9,10). Both patients were found to have multiple cerebral metastases in different areas of the brain several months after their initial diagnosis of anal cancer. Both patients passed away before beginning the planned radiotherapy. In total, 3 patients succumbed to the disease either during the treatment of the brain metastasis or shortly after completing secondary treatment (8,10,13). All cerebral metastases were treated or planned for treatment with surgical resection, radiation therapy or a combination of the two. No case report documented the use of chemotherapy in treating cerebral metastasis. In a review by Sclafani *et al* (17) in 2019, treatment protocols in 8 reports of advanced anal SCC with liver and lung metastasis were compared and it was found that although there is a lack of high-level evidence to guide the treatment of advanced anal SCC, a multi-disciplinary approach to treating metastatic disease was considered a superior approach. It was concluded that aggressive management with various treatment modalities, including surgery, radiotherapy and radiofrequency ablation, can offer more extended disease control compared with systemic chemotherapy alone. However, given the rarity of the disease and lack of supportive evidence, further randomised control trials are urgently necessary to develop a substantial treatment regime tailored to individualised treatments.

A case reported in 2017 by Ogawa *et al* (7) documented the longest survival period from diagnosis of primary malignancy to death, which was 5 years. The case involved a female patient in her 60s who was initially diagnosed with poorly differentiated SCC, which was positive for cytokeratin (CK) 5, 6 and 34bE12, and negative for CDX2. CK5 and 6 are understood to be indicative of squamous cell origin, ruling out adenocarcinoma and neuroendocrine origins (18). At the initial presentation, the patient also had liver and internal iliac lymph node involvement. Initial treatment included CRT with 5-FU and cisplatin with 54 Gy over 27 fractions of radiotherapy. However, 7 months after initial diagnosis, metastatic brain lesions were found in the occipital lobe and cerebellum. The patient received 19 Gy of radiation to the occipital lesion and 21 Gy to the cerebellar lesion. Over the next 4 years, the patient experienced multiple recurrences of the primary cancer, as well as further cerebral metastasis. Despite receiving various treatments, including radiation, surgical resection and chemotherapeutics, the patient experienced significant adverse events, including significant anorexia and ceased further treatment after 5 years (7).

The present patient's primary SCC displayed strong, diffuse cytoplasmic and nuclear labelling for p16. The only other documented case of p16 positivity was reported by Malla *et al* (6). p16 upregulation was previously considered an indicator of HPV-induced transformation to anal carcinoma; however, it has since been discovered to occur independent of HPV infection. HPV-negative tumours more frequently correlate with TP53 mutations compared with p16 upregulation (19), while p16 upregulation can occur independent of HPV via the p16/ retinoblastoma protein (Rb) pathway. Specifically, if p16 expression is lost, unregulated CDK4/6 expression leads to hyperphosphorylated Rb, ultimately leading to uncontrolled cell proliferation (20). p16 has also been used as a marker of malignancy in cervical SCC. It was found that the intensity with which p16 stained immunohistochemically has a direct relationship to the severity of cervical lesion or disease (21).

HPV and p16 should only be considered in tandem; p16 independently is not considered to be a reliable marker of HPV-induced transformation as p16 can be positive for malignancies unrelated to HPV (22). Although not an exhaustive list, p16 overexpression is seen in endometrial carcinoma (23) and mesothelioma (24). The presence of p16 positivity without HPV in anal SCC tends to lead to a poorer prognosis due to a reduced response to treatment (14). The presence of both HPV and p16 is linked to an increased response to treatment, whereas HPV and p16 negative status records the lowest rate of response to treatment and the worst prognosis overall (14). A meta-analysis in 2017 concluded that patients with HPV+/p16+ status anal SCC had improved outcomes than those with HPV-/p16+ or HPV-/p16-status. Additionally, it was suggested that an individual's HPV and p16 status can be used for therapeutic guidance and of prognostic utility in anal SCC (25). The American Society of Colon and Rectal Cancer Surgeons guidelines suggests that there may be benefit in vaccination against HPV to prevent anal cancer altogether (26). The present patient did not have any risk factors for anal cancer other than smoking. At the time of initial diagnosis of anal SCC, the patient had been a smoker for a couple of decades, although had since ceased smoking.

Patients who complete CRT may benefit from assessment of clinical response to the primary malignancy at 6 months with a digital rectal exam, palpation of inguinal lymph nodes, and a yearly CT/positron emission tomography scan for up to 3 years (27,28). However, no significant data supports investigating distant metastasis during the surveillance period (27). The benefits of full-body scanning in patients who present with evidence of liver and/or lung metastasis at the initial diagnosis of an anal primary should be considered. This does, however, carry implications. For example, the cost-effectiveness of a full-body scan, as well as the availability of such scans. Furthermore, a false positive can inflict unnecessary distress on a patient and their family members. Other logistical barriers also exist, including insurance limitations and transport to such facilities. Further research aiming to optimise surveillance may potentially benefit gastrointestinal malignancies altogether.

Anal SCC with metastatic spread to the brain presents a poor prognosis as survival from the time of discovery of cerebral lesions to death is a short few months to years (5). Distant spread to the brain follows no understood pattern concerning either location, quantity or timeline. Additionally, those who present without metastasis initially do not necessarily carry an improved prognosis when compared with those who present with metastasis. The case report by Davidson and Young was the first to report that their patient had been in remission of their primary anal SCC at the time of presentation with cerebral disease (11). This implies that at the time of declaring remission, there is a likelihood that microscopic malignant cells will linger undetected at the time of a follow-up scan. Colorectal cancer may spread to the liver via the portal circulation, which can lead to hepatic metastasis while increasing the risk of spread to the lungs as well. From here, a highway is created for dissemination of metastases to the central nervous system (29).

Additionally, a brain lesion may then be missed in the workup and surveillance for anal cancer as the brain is not routinely imaged unless otherwise clinically indicated (30). It may be beneficial to consider a CT of the brain with contrast in patients with initial metastasis in the liver and/or lungs to rule out cerebral metastasis at presentation confidently.

Furthermore, utilisation of a chemotherapeutic agent that can effectively cross the blood-brain barrier (BBB) could be considered, to ensure that spread to the brain is minimised or prevented in patients with known existing metastatic disease. For example, an agent such as temozolomide, which has historically been used in treating gliomas and can penetrate the BBB, may be considered in preventing cerebral metastasis (31). A review of 21 clinical trials in 2014 investigated the efficacy of temozolomide in treating cerebral metastases from various solid tumours which included non-small cell lung cancer and malignant melanoma. It was noted that trials with temozolomide-monotherapy had modest response, while metastases treated with combination chemotherapy with temozolomide and radiation therapy saw an even greater response to treatment compared with monotherapy alone (32).

Of the previous cases reported, no two primary anal malignancies nor cerebral metastasis were treated identically. Primary treatment consisted of either surgery, chemotherapy, radiation or a combination of the three. This area of gastrointestinal malignancies would benefit from further research in standardising primary treatment to decrease the risk of recurrence and metastatic spread and develop treatment protocols for advanced disease and surveillance protocols. Whether investigating cerebral lesions before declaring remission would be of benefit warrants further study. Finally, testing for tumour markers such as p16 may be considered when planning treatment and to further understand the disease course. In conclusion, patients at risk for anal cancer should take steps to protect their health by mitigating risks and undergoing regular screening to detect any potential problems early.

## Figures and Tables

**Table I.** Previously reported cases of anal squamous cell carcinoma.

First author, year	Age, years	Sex	Histopathology	Location of distant metastasis at time of diagnosis	Initial treatment	Time from diagnosis to cerebral metastasis	Location of cerebral metastasis and subsequent treatment	Outcome	(Refs.)
Klotz *et al*, 1967	Information not provided.	Information not provided.	Transitional cloacogenic carcinoma (subtype of squamous cell carcinoma)	Nil	Information not provided.	Information not provided.	Information not provided.	Information not provided.	(12)
Davidson *et al*, 1991	53	F	Basaloid carcinoma (subtype of SCC)	Nil	Abdominoperineal surgery.	8 years	Cerebellum. Surgical excision and palliative radiotherapy.	Succumbed to disease 3 months after diagnosis of cerebral metastasis.	(11)
Rughani *et al*, 2011	63	F	Poorly differentiated SCC	Inguinal lymphadenopathy and liver lesion.	17 doses of pelvic radiation with oral chemotherapy (capecitabine 1500 mg, twice daily).	Unclear	Right parietal lobe lesion. Elective craniotomy and whole brain radiation.	Neurological symptoms unchanged at 1-month post-operative follow up. Patient succumbed to disease 14 weeks post diagnosis of cerebral metastasis.	(10)
Gassman *et al*, 2012	67	M	Invasive poorly differentiated SCC.	Isolated hepatic metastasis.	5-FU, cisplatin + 50.4 Gy radiation, followed by further cisplatin therapy.	6 months	Intracranial mass with involvement of the sphenoid and temporal bones, in addition to left orbit, CNII and rectus muscle. Stereotactic radiotherapy planned for newly found brain metastasis.	Patient experienced recurrence of primary disease, treated with abdominoperineal and liver resection for recurrence. Patient succumbed to disease prior to commencement of stereotactic radiotherapy for brain metastasis.	(9)
Hernando-Hubero *et al*, 2014	69	M	Stage IV basaloid undifferentiated carcinoma.	Mesorectal lymphadenopathy, multiple pulmonary and liver metastases.	Cisplatin + 5-FU	14 months	Right parietal, left rolandic, cortical and subcortical, cerebellar and frontal lobes, with tonsillar herniation. Steroid therapy + 30 Gy.	Patient succumbed to disease 12 weeks after brain metastasis discovered.	(13)
Ogawa *et al*, 2017	60′s	F	Poorly differentiated SCC, positive for CK5/6 and 34βE12, and negative for CDX2. Negative for HPV and p16.	Liver and internal iliac lymph node.	Two cycles of 5-FU 700 mg/m^2^ and cisplatin 70 mg/m^2^ + 54 Gy/27 fractions of radiotherapy.	7 months	Metastatic lesions in occipital lobe (19 Gy) and cerebellum (21 Gy). Patient then underwent surgical excision of occipital tumour that had grown, while cerebellar lesion showed full response to treatment. A fluoropyrimidine anticancer agent was administered + 22 Gy for progression of liver metastasis. Occipital tumour recurrence detected, subsequently treated with 22 Gy of radiotherapy. Recurrence of occipital lesion was treated surgically.	Patient experienced adverse effects from treatment which included significant anorexia. After 5 years, treatment was ceased. Out-patient follow up was scheduled. Longest reported survival of a patient with a distant cerebral metastasis.	(7)
Chihabeddine *et al*, 2021	44	F	Poorly differentiated SCC	Extension into gluteal soft tissue and involvement of mesorectal nodules, cutaneous nodules of the sacral region, and bone lesions of greater trochanter.	Chemotherapy with capecitabine 825 mg/m^2^, cisplatin 80 mg/m^2^ + radiotherapy total dose of 60 Gy.	A few months	Left parietal lobe and frontal bone with involvement of soft tissue of scalp. Total brain external radiotherapy, total dose of 20 Gy.	Patient succumbed to disease during treatment.	(8)
Chihabeddine *et al*, 2021	60	M	Moderately differentiated SCC with positive anti p40. Negative for programmed death ligand 1 and microsatellite instability.	Secondary lung localization, external iliac and inguinal lymphadenopathy. Lesions seen in sacrum, pubis, and coccyx.	Palliative chemotherapy docetaxel, cisplatin, FU + granulocyte colony-stimulating factor, docetaxel, and 5-FU for 12 weeks. Treatment ceased early due to persistent neutropenia. Second-line treatment: 60 Gy, capecitabine and cisplatin.	A few months	Multiple metastatic lesions above and below the tentorium Total brain radiotherapy planned.	Patient succumbed to disease prior to starting treatment.	(8)
Malla *et al*, 2022	54	F	Poorly differentiated SCC with ki-67 at 80%. Positive for p63 and pancytokeratin. p16 also strongly positive.	Nil	5-FU and mitomycin	3 months	Right frontal mass with significant oedema. Surgical resection and g knife radiosurgery. Whole brain radiotherapy with 3,000 Gy and intensity modulated radiation therapy.	Patient experienced recurrence 8 months later and had another round of g knife radiosurgery. Following another recurrence of a brain lesion 7 months later, patient opted for hospice care.	(6)

F, female; M, male; HPV, human papilloma virus; SCC, squamous cell carcinoma; 5-FU, 5-fluorouracil.

## Data Availability

The data generated in the present study are not publicly available due to patient confidentiality.
